# Abnormal Spontaneous Neural Activity in Obsessive-Compulsive Disorder: A Resting-State Functional Magnetic Resonance Imaging Study

**DOI:** 10.1371/journal.pone.0067262

**Published:** 2013-06-27

**Authors:** Li Ping, Li Su-Fang, Han Hai-Ying, Dong Zhang-Ye, Luo Jia, Guo Zhi-Hua, Xiong Hong-Fang, Zang Yu-Feng, Li Zhan-Jiang

**Affiliations:** 1 Department of Psychiatry, Qiqihaer Medical University, Qiqihaer, Heilongjiang, China; 2 Department of Psychiatry and Clinical Psychology, Beijing Anding Hospital, Capital Medical University, Beijing, China; 3 State Key Laboratory of Cognitive Neuroscience and Learning, Beijing Normal University, Beijing, China; 4 Center for Cognition and Brain Disorders and the Affiliated Hospital, Hangzhou Normal University, Hangzhou, Zhejiang, China; Emory University, United States of America

## Abstract

Neuroimaging studies of obsessive-compulsive disorder have found abnormalities in orbitofronto-striato-thalamic circuitry, including the orbitofrontal cortex, anterior cingulate cortex, caudate, and thalamus, but few studies have explored abnormal intrinsic or spontaneous brain activity in the resting state. We investigated both intra- and inter-regional synchronized activity in twenty patients with obsessive-compulsive disorder and 20 healthy controls using resting-state functional magnetic resonance imaging. Regional homogeneity (ReHo) and functional connectivity methods were used to analyze the intra- and inter-regional synchronized activity, respectively. Compared with healthy controls, patients with obsessive-compulsive disorder showed significantly increased ReHo in the orbitofrontal cortex, cerebellum, and insula, and decreased ReHo in the ventral anterior cingulate cortex, caudate, and inferior occipital cortex. Based on ReHo results, we determined functional connectivity differences between the orbitofrontal cortex and other brain regions in both patients with obsessive-compulsive disorder and controls. We found abnormal functional connectivity between the orbitofrontal cortex and ventral anterior cingulate cortex in patients with obsessive-compulsive disorder compared with healthy controls. Moreover, ReHo in the orbitofrontal cortex was correlated with the duration of obsessive-compulsive disorder. These findings suggest that increased intra- and inter-regional synchronized activity in the orbitofrontal cortex may have a key role in the pathology of obsessive-compulsive disorder. In addition to orbitofronto-striato-thalamic circuitry, brain regions such as the insula and cerebellum may also be involved in the pathophysiology of obsessive-compulsive disorder.

## Introduction

Obsessive-compulsive disorder (OCD) is a chronic anxiety disorder marked by recurrent, intrusive, and distressing thoughts (obsessions) and/or repetitive behaviors (compulsions) [1]. Although the pathophysiology of OCD remains unclear, neuroimaging studies have reported abnormalities in brain structure and function [2], [3]. Most abnormalities have been reported in orbitofronto-striato-thalamic circuitry, including the orbitofrontal cortex (OFC), anterior cingulate cortex (ACC), caudate, and thalamus [2], [4], [5], [6], [7], [8]. In contrast, a recent neuroimaging study suggested that the pathophysiology of OCD may involve more widely distributed large-scale brain systems, including the parietal and occipital lobes and the cerebellum, rather than the conventional orbitofronto-striatal model [9]. However, previous studies of dysfunctional brain activity in OCD used different cognitive tasks, which may have contributed to the absence of consistent and compelling conclusions. It also is unknown whether these brain areas have abnormal intrinsic or spontaneous brain activity in the resting state.

Resting-state functional magnetic resonance imaging (RS-fMRI) has been successfully applied to many neurological disorders [10]. During RS-fMRI scanning, participants are instructed to relax and not engage in any cognitive processes. Therefore, the signals reflect spontaneous or intrinsic brain activity [11], [12].

Recent RS-fMRI studies of OCD have reported compelling results. Harrison et al (2009) tested the strength of functional connectivity in four striatal seed regions of interest (ROIs). They reported that patients with OCD had increased functional connectivity along a ventral cortico-striatal axis that includes the OFC, and the strength of functional connectivity between the ventral caudate and OFC correlated with the severity of the patients’ symptoms [13]. Sakai et al. (2010) found positive functional connectivity between the ventral striatum and the OFC, dorsolateral prefrontal cortex, and ventral medial prefrontal cortex was significantly stronger in non-medicated patients with OCD than in controls [14]. The above studies suggest that the OFC may have a crucial role in OCD. However, as most studies, including those our group has conducted, used functional connectivity to measure correlations between the time series of distinct brain regions [13], [14], [15], [16], [17], [18], the resulting abnormal functional connectivity cannot determine the specific brain region that is exhibiting abnormal activity.

Regional homogeneity (ReHo) is a method for measuring local synchronization of spontaneous activity within neighboring voxels in the resting state. Increased and decreased ReHo may be related to disequilibrium of baseline activity in a brain region, and may be related to dysregulated mood and behavior, and dysregulated communication between corresponding brain regions [12]. ReHo analysis has been successfully used to detect brain dysfunctions in many diseases, such as schizophrenia, depression, and autism [19], [20], [21].

Previous studies strongly suggest that the OFC has a crucial role in the pathophysiology of OCD [2], [3]. We hypothesized that patients with OCD have abnormal local synchronization in the OFC, and the OFC will show abnormal functional connectivity in patients with OCD. The aim of the present study was to investigate both intra-regional synchronized activity (i.e., ReHo) and inter-regional synchronized activity (i.e., functional connectivity) in patients with OCD using RS-fMRI. In addition, we examined the correlation between abnormal brain activities and the clinical symptoms of OCD.

## Materials and Methods

### Participants

Twenty-five outpatient subjects with OCD were recruited at Beijing Anding Hospital, Capital Medical University. The Anxiety Disorders Interview Schedule for DSM-IV: Lifetime Version (ADIS-IV-L) [22] was used to diagnose OCD and exclude other anxiety disorders. The Yale-Brown Obsessive-Compulsive Scale (Y-BOCS) [23], the 17-item Hamilton Depression Rating Scale (HAMD-17) [24] and the Hamilton Anxiety Rating Scale (HAMA) [25] were used to rate the severity of obsessive-compulsive, depression, and anxiety symptoms, respectively. Only patients with a score of 16 or more on the Y-BOCS scale and a score less than 18 on the HAMD-17 scale were included. Other inclusion criteria we used were being between 18 and 50 years old and right-handed. The exclusion criteria we used were a history of neurological illness or other major physical diseases, a history of psychiatric disorders such as schizophrenia or mood disorders, or a history of psychoactive substance or alcohol dependence and/or abuse.

Data from five patients were excluded because of excessive head movement during the fMRI acquisition (see image preprocessing). For the remaining twenty patients, two had never taken any medications for OCD, four had not taken medications for OCD in the past month, and fourteen were on stable doses of serotonin reuptake inhibitors (SRIs) at the time of the scan. Twenty healthy volunteers were recruited from the local community as assessed using the Structured Clinical Interview for DSM-IV Axis I Disorders-Non-patient Edition (SCID-I/NP) [26]. None of the healthy controls had any neurological illness, major physical diseases, psychiatric disorders, or a positive family history of major psychiatric disorders. The twenty case-control pairs were matched for age, sex, handedness, and years of education (Table 1). This study was approved by the Research Ethics Committee at Beijing Anding Hospital, Capital Medical University. Written informed consent was obtained from all participants.

**Table 1:** Demographic and clinical data of the participants.

	OCD patients(n = 20)	Healthy controls(n = 20)	*P-*Value
Age (years)	27.1±8.0	27.6±8.2	0.821
Sex (male/female)	16/4	16/4	
Education (years)	14.2±2.1	14.0±2.3	0.777
Illness duration (months)	88.1±69.6		
Y-BOCS, Total score	23.5±5.9		
Obsession score	14.5±3.4		
Compulsion score	9.1±4.8		
HAMD total score	11.2±6.4		
HAMA total score	12.9±6.9		

Data are presented as mean ± standard deviation or number.

OCD: obsessive compulsive disorder. Y-BOCS: Yale-Brown Obsessive-Compulsive Scale. HAMD: 17-item Hamilton Depression Rating Scale. HAMA: Hamilton Anxiety Rating Scale.

### Image Acquisition

Functional images were obtained on a Siemens Trio 3-tesla scanner at the State Key Laboratory of Cognitive Neuroscience and Learning, Beijing Normal University, Beijing, China. Subjects lay supine with foam pads and earplugs to reduce head motion and scanner noise. The scanning sessions included localization, RS-fMRI, 3D T1 anatomical (not used in the current study), diffusion tensor imaging (not used in the current study), and conventional T2 imaging sequences. Resting-state was defined as a subject not engaging in any specific cognitive task during fMRI scanning [27]. During the RS-fMRI acquisition, subjects were instructed to hold still, relax, close their eyes, avoid falling asleep, and to not think of anything in particular. The resting-state functional scans were obtained using an echo-planar imaging (EPI) sequence with the following parameters: 33 axial slices, TR = 2000 ms, TE = 30 ms, FA = 90°, thickness/gap = 3.5/0.6 mm, FOV = 200×200 mm, in-plane resolution = 64×64, and totally 240 volumes (8 minutes). No subject showed any structural abnormalities on visual inspection. It should be noted that the scanner we used did not have any technique to prevent the ventromedial prefrontal drop-out from sinus artifact.

### Image Preprocessing

Image preprocessing was carried out using the Data Processing Assistant for RS-fMRI (DPARSF, http://www.restfmri.net) [28], which is based on Statistical Parametric Mapping (SPM5) (http://www.fil.ion.ucl.ac.uk/spm) and RS-fMRI Data Analysis Toolkit (REST) [29]. The first 10 time points were discarded for signal stabilization and to allow for the adaptation of the participants to the situation. Slice timing and head motion correction were conducted. Five patients with OCD were excluded from further analysis because of excessive head motion (more than 2 mm of translation or more than 2 degrees of rotation in any direction). The functional images were normalized using the standard EPI template in SPM, and spatially resampled to a voxel size of 3×3×3 mm^3^. Linear trends were removed. A band-pass (0.01–0.08 Hz) filter was applied to reduce low-frequency drifts and physiological high frequency respiratory and cardiac noise [27].

### ReHo Analysis

RS-fMRI data without spatial smoothing were used for the ReHo analysis using DPARSF (http://www.restfmri.net) [28]. Kendall's coefficient of concordance (KCC) was calculated to measure the local synchronization of 27 nearest neighboring voxels [12], and the ReHo value was assigned to the central voxel. Then a ReHo map was obtained in a voxel-wise manner. Thus, each individual ReHo map was generated. A standardized ReHo map was created by dividing every individual ReHo map by each participant's global mean KCC value within the brain mask. Finally, the standardized ReHo maps were spatially smoothed with a Gaussian kernel (FWHM) of 4 mm. It has been noted that spatial smoothing before ReHo calculation will dramatically increase the ReHo value [28]. An independent two-sample t-test was performed on the standardized ReHo maps with SPM5. Voxels with a *p* value less than 0.05 and a cluster size greater than 1566 mm^3^ (54 voxels) were considered significantly different, corresponding to a corrected *p* value less than 0.05 as determined by AlphaSim in the Analysis of Functional Neuroimages software package (http://afni.nimh.nih.gov/pub/dist/doc/manual/AlphaSim.pdf).

### Functional-connectivity Analysis

Brain regions satisfying the threshold described above were analyzed for functional connectivity. For each region, a spherical ROI with a 4-mm radius was centered at the voxel that showed the most significant between-group difference within that region. Brain regions in the OFC (See results for details) that showed significant between-group differences were taken as seed regions and the functional-connectivity analysis was performed between these ROIs in the OFC and the remaining ROIs. We used nine nuisance covariates that include the six head-motion parameters, the global mean timecourse, white matter time course, and cerebrospinal fluid time course. Fisher’s *z* transformation was used to improve the normality of the correlation coefficients [30], [31], and the resulting correlation coefficients were transformed into *z* values. All the above procedures were executed using DPARSF (http://www.restfmri.net) [28]. Recently, Functional connectivity analysis with global mean timecourse removal has been demonstrated that it can artificially induce negative functional correlations [32]. Thus, we also run the functional connectivity analysis between OFC and all other ROIs without regressing out the global mean timecourse.

### Statistical Analysis

In addition to the statistical analysis described above, other statistical analyses were performed using the SPSS 13.0 software package (SPSS Inc., Chicago, Illinois, USA). Independent t*-*tests were performed on the demographic and clinical data and the functional connectivity *z-*values. The significance level was set at 0.05. We used Pearson’s correlation analysis to assess the relationship between the RS-fMRI measures (i.e., ReHo values and the strength of the functional connectivities from the connections that showed significant group differences) and the clinical presentation of the subjects, including the severity of obsessive-compulsive symptoms and the OCD duration.

## Results

### Demographic and Clinical Data

Demographic and clinical data are shown in Table 1. There were no significant differences in age and years of education (all *p*>0.05) between the OCD group and the control group.

### ReHo Analysis between Patients with OCD and Healthy Controls

**Figure 1:**
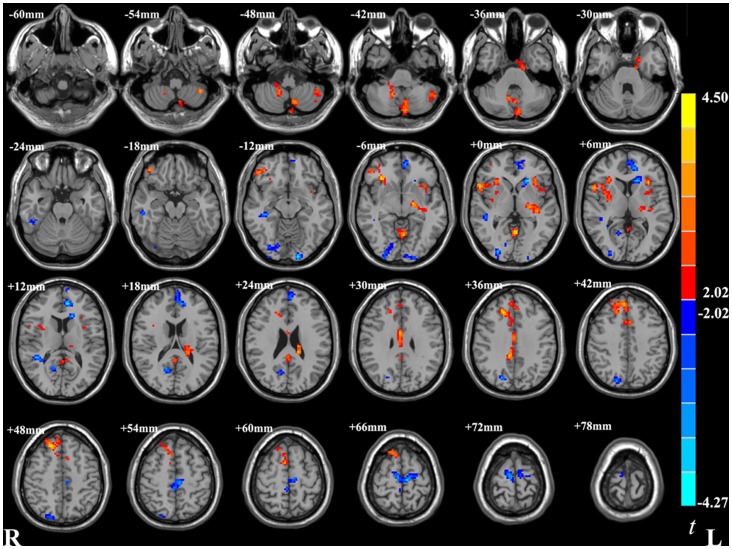
Significant different ReHo in OCD group than healthy controls group in a whole brain analysis. These maps are the results of an independent two-sample t-test with *p*<0.05 and cluster size >1566 mm^3^. R: right side. L: left side. Red and blue denote increased and decreased ReHo respectively. The color bar indicates the t-values.

As shown in Figure 1 and Table 2, compared with the healthy controls, patients with OCD showed increased ReHo in the right OFC, bilateral insula, right superior frontal gyrus, right middle cingulate cortex, and the bilateral cerebellum. We observed decreased ReHo in the patients with OCD in the left ventral ACC (vACC), left caudate, right inferior occipital cortex (IOC), left lingual gyrus, right calcarine, right cuneus, and right supplementary motor area.

**Table 2:** Brain regions showing significant (corrected) difference in ReHo between patients with obsessive compulsive disorder and controls.

Hemisphere	Region	Brodmann’ area	Number of voxels	Coordinates of peak voxel	t value of peak voxel
**Increased ReHo in OCD**					
L	white matter**^1^**		54	−12, −12, −33	3.10
L	cerebellum		73	−39, −51, −54	3.37
L	cerebellum		155	−6, −81, −42	3.48
R	OFC-1	47	74	48, 39, −9	3.56
R	cerebellum		71	15, −54, −45	3.64
R	MCC	24	79	3, −3, 30	3.74
L	insula	48	99	−33, 21, 3	3.76
L	cerebellum		160	−3, −60, −3	3.86
L	white matter**^2^**		183	−15, −33, 21	3.94
R	insula	47	200	33, 27, −6	4.12
R	SFG	32	400	18, 30, 36	4.32
**Decreased ReHo in OCD**					
R	white matter**^3^**		54	39, −30, −12	−3.35
R	Calcarine	17	61	15, −60, 15	−3.43
R	Cuneus	19	74	18, −78, 39	−3.62
R	IOC	18	127	27, −90, 0	−3.65
R	white matter**^4^**		77	42, −42, 12	−3.73
L	vACC	32	209	−9, 45, 12	−4.01
R	SMA	6	253	6, −18, 69	−4.12
L	caudate	47	82	−21, 24, 0	−4.14
L	Lingual	18	59	−15, −99, −12	−4.27

R: right. L: left. OFC: orbital frontal cortex. SFG: superior frontal gyrus. MCC: middle cingulate cortex. vACC: ventral anterior cingulate cortex. IOC: inferior occipital cortex. SMA: supplementary motor area. 1: This cluster is close to the left parahippocampal gyrus; 2: This cluster is close to the left thalamus and caudate; 3: This cluster is close to the right hippocampus; 4: This cluster is close to the right superior temporal gyrus.

### Functional Connectivity

**Figure 2:**
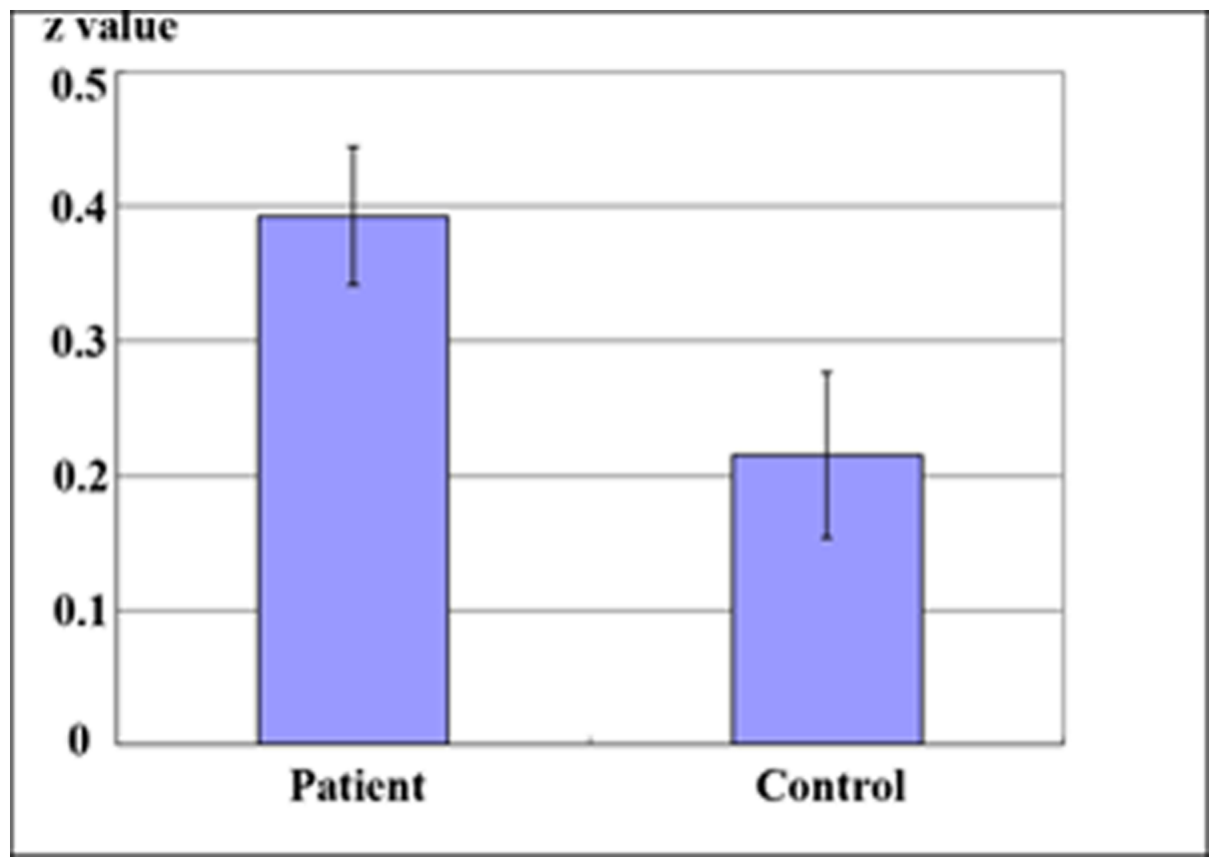
Significant between-group differences in functional connectivity between OFC and ACC. These maps are the results of an independent two-sample t-test with *p*<0.05. Patients with OCD demonstrated increased positive functional connectivity between OFC and the left vACC. Bars and error bars represent the mean and standard deviation of *Z* value in OCD group and healthy controls group respectively.

Given that the OFC has been consistently found to be abnormal in patients with OCD [2], [3], in this study, we were interested not only in local abnormalities of the OFC but also in its functional connectivity. Hence, the OFC that showed increased ReHo in patients with OCD were taken as seed ROI. Functional connectivity-analysis was performed between OFC and all other ROIs showing significant ReHo differences between patients with OCD and control subjects (Figure 1 and Table 2). Compared with healthy controls, patients with OCD demonstrated increased positive functional connectivity between OFC and the left vACC when regressing out the six head-motion parameters, the global mean timecourse, white matter timecourse, and cerebrospinal fluid timecourse (Figure 2 and Table 3). However, when the global mean timecourse was not regressed out (others were kept the same as motioned above), there was no significant functional connectivity difference between the OFC and other ROIs in OCD compared with healthy controls (Table 4).

**Table 3:** Strength (*Z* value, mean and standard deviation) of the functional connectivity between OFC and the other ROIs with removing global mean timecourse and between group difference (*P* value of two-sample t-test).

ROI	Patients	Controls	*P* Value
vACC	0.39±0.23	0.22±0.27	0.03
MCC	0.43±0.36	0.28±0.24	0.11
Cuneus	0.34±0.27	0.22±0.22	0.14
caudate	0.14±0.16	0.24±0.26	0.16
Insula (−33, 21, 3)	0.29±0.30	0.36±0.31	0.48
Cerebellum (−6, −81, −42)	0.31±0.30	0.27±0.23	0.63
IOC	0.21±0.25	0.17±0.28	0.64
Insula (33, 27, −6)	0.45±0.26	0.41±0.28	0.67
Cerebellum (−3, −60, −3)	0.28±0.35	0.24±0.29	0.68
Lingual	0.28±0.21	0.26±0.27	0.81
SMA	0.23±0.30	0.21±0.22	0.82
Cerebellum (15, −54, −45)	0.14±0.46	0.12±0.21	0.87
SFG	0.17±0.26	0.16±0.19	0.89
Calcarine	0.17±0.40	0.15±0.32	0.89
Cerebellum (−39, −51, −54)	0.29±0.38	0.29±0.21	0.99

SFG: superior frontal gyrus. MCC: middle cingulate cortex. vACC: ventral anterior cingulate cortex. IOC: inferior occipital cortex. SMA: supplementary motor area.

**Table 4:** Strength (*Z* value, mean and standard deviation) of the functional connectivity between OFC and the other ROIs without removing global mean timecourse and between group difference (*P* value of two sample t-test).

ROI	Patients	Controls	*P* Value
MCC	0.43±0.41	0.23±0.27	0.08
caudate	0.11±0.19	0.24±0.31	0.12
vACC	0.37±0.29	0.23±0.31	0.15
Insula (−33, 21, 3)	0.26±0.30	0.36±0.32	0.31
Cerebellum (−6, −81, −42)	0.33±0.43	0.23±0.22	0.32
Lingual	0.29±0.25	0.37±0.32	0.39
Cerebellum (−3, −60, −3)	0.33±0.37	0.24±0.32	0.44
Cuneus	0.32±0.29	0.26±0.25	0.47
Cerebellum (15, −54, −45)	0.17±0.48	0.08±0.29	0.47
Cerebellum (−39, −51, −54)	0.28±0.43	0.33±0.16	0.64
IOC	0.25±0.32	0.29±0.33	0.69
Calcarine	0.21±0.41	0.16±0.36	0.69
SMA	0.27±0.28	0.25±0.26	0.77
SFG	0.22±0.34	0.20±0.19	0.81
Insula (33, 27, −6)	0.44±0.26	0.45±0.30	0.94

SFG: superior frontal gyrus. MCC: middle cingulate cortex. vACC: ventral anterior cingulate cortex. IOC: inferior occipital cortex. SMA: supplementary motor area.

### Association between the Clinical Presentation of OCD with ReHo and the Strength of Functional Connectivity

A positive correlation was found between the ReHo of the caudate and OCD duration (r = 0.544, *p* = 0.019), and an inverse correlation was found between the ReHo of the OFC (x, y, z = 48, 39, −9) and OCD duration (r = −0.489, *p* = 0.039). An inverse correlation was also found between the ReHo of the cerebellum (x, y, z = 15, −54, −45) and the Y-BOCS compulsive scores (r = −0.496, *p* = 0.037). No significant correlation was observed between the strength of functional connectivity (OFC - vACC) that showed significant group differences and OCD duration (r = 0.368, *p* = 0.146 ), Y-BOCS total scores (r = −0.200, *p* = 0.441), obsession scores (r = −0.249, *p* = 0.334), or compulsive scores (r = −0.089, *p* = 0.735). The analysis controlled for the patients’ comorbid depression and anxiety ratings on the HAMA and HAMD-17.

## Discussion

In this study, we combined ReHo and functional connectivity methods to analyze RS-fMRI data. We found that patients with OCD showed abnormal spontaneous neural activities in the OFC, vACC, caudate, cerebellum, insula, and occipital cortex using the ReHo method. Furthermore, the vACC showed abnormal functional connectivity with OFC in patients with OCD. After controlling for comorbid depression and anxiety, the ReHo of the OFC and caudate were correlated with the OCD duration. The ReHo of the right cerebellum was associated with the severity of compulsive symptoms.

### Abnormal Intra-regional Synchronized Activity (ReHo) in OCD

Compared with healthy controls, patients with OCD showed higher ReHo in the right OFC. ReHo reflects the level of coordination of regional neural activity in the resting state. Functional brain imaging studies have demonstrated significantly increased OFC activity in both the resting state [2] and during active symptoms of OCD [33], [34], [35] and decreased activation during cognitive tasks [4], [5], [36]. The OFC has an important role in response inhibition and behavior suppression [37], [38]. Many studies reported that patients with OCD and their unaffected first-degree relatives have deficits in response inhibition, suggested the deficit in response inhibition may be a candidate endophenotype for OCD [39], [40], [41]. It is possible that increased local synchronization in the baseline activity of the OFC may contribute to poor inhibitory control in patients with OCD, and result in recurrent thoughts, intrusive thoughts, and repetitive behaviors. Moreover, the ReHo of the OFC was negatively correlated with the OCD duration, which suggests the OFC may be a marker for the progression of OCD.

There was decreased ReHo in the vACC in patients with OCD in the current study. Two studies have found the vACC shows increased activity during the symptoms of OCD [33], [35]. Contributions of the vACC have been implicated in assessing the significance of emotional information and the regulation of emotional responses [42], [43]. Thus, disequilibrium of baseline activity in this region may be related to maladaptive emotional responses (i.e., anxiety and depression) in patients with OCD.

In line with some positron emission tomography (PET) and single photon emission computed tomography (SPECT) studies [44], there was decreased ReHo in the caudate in patients with OCD. Abnormalities in the caudate have been consistently reported in OCD [44] and may be an OCD-specific feature [45]. As the caudate is involved in selecting and producing new activity patterns [46], the decreased resting-state local synchronization in this region in patients with OCD may indicate a decreased ability to react flexibly to new information. Moreover, abnormal activity in the caudate can affect the OFC via a subcortical-cortical loop [46].

Although previous resting-state PET studies have found increased glucose metabolism in the caudate in patients with OCD [3], we found decreased ReHo in the caudate. This discrepancy may be because of several reasons. First, treatment with SRIs can normalize higher regional brain activity of the caudate [47], [48]. SRIs may also affect the local synchronization of the caudate, thus decreasing the ReHo of the caudate. Second, although higher ReHo in the default-mode network during resting-state indicates higher brain activity [12], which is a similar pattern to a resting-state PET study [49], there has not been a study on the correlation between ReHo and glucose metabolism in a same group of subjects. The relationship between local synchronization and local brain metabolism needs to be investigated.

In the present study, we found higher ReHo in bilateral insula, a region of the paralimbic system that is involved in mediating emotional states such as anxiety [50]. Increased activation in the insula during active symptoms of OCD has been reported [33], [45]. Moreover,Lázaro et al. (2008) found significantly decreased activation in the left insula after 6 months of treatment with a selective serotonin reuptake inhibitor and improved clinical symptoms in patients with OCD [51]. Increased local synchronization of resting-state spontaneous activity in the insula may suggest that higher emotional reactivity of patients with OCD is the result of increased baseline activity in the insula.

In the current study, we found increased ReHo in the cerebellum. Many studies indicate the cerebellum may be involved in OCD [2], [34], [52]. The cerebellum is a part of numerous cognitive networks and may have a role in some cognitive functions, including language, working memory, spatial processing, and executive function [53]. The increased local synchronization of baseline activity in the cerebellum, therefore, may contribute to some symptoms of OCD, such as poor inhibitory control and repetitive behavior. This may have relevance for our findings of a significant correlation between baseline activity in the cerebellum and the severity of compulsive symptoms.

### Abnormal Inter-regional Synchronized Activity (Functional Connectivity) in OCD

In this study, we investigated both local or intra-regional synchronized activity within the OFC and its inter-regional synchronized activity (i.e., functional connectivity) with other brain regions we found to have abnormal ReHo. Functional connectivity is a temporal correlation between remote brain areas [54], and positive functional connectivity may have an integrative role in combining neuronal activity for similar goals [55]. In OCD, there is increased positive functional connectivity between the OFC and the vACC when the global mean timecourse was regressed out, which may contribute to the clinical presentation of the failure to inhibit automatic thoughts and repeated behaviors [56]. This behavior often then leads to anxiety and depression. There is no significant functional connectivity difference between the OFC and other ROIs in OCD compared with healthy controls when the global mean timecourse was not regressed out. Global mean timecourse removal in functional connectivity analysis may artificially cause spurious findings of negatively correlated regions in the brain [32]. In our study, we performed ROI-wise functional connectivity analysis and did not find negative functional connectivity between the OFC and any other region showing significant ReHo differences between two groups. It should be noted that, among the total 30 pairs of functional connectivity (Table 3+ Table 4), there was only one with significant group-difference. It does not survive multiple comparison correction. Therefore, the interpretation of the current abnormal functional connectivity in OCD should be cautious.

### Limitations

There are several limitations of the current study. First, 14 patients in the OCD group were taking stable doses of SRIs at the time of the scan. Therefore SRIs effects on intra- and inter-regional synchronized activity cannot be excluded, especially considering that SRIs can alter abnormal brain function and metabolism in patients with OCD [51], [57]. Future studies should examine the effects of SRIs on spontaneous brain activity using RS-fMRI. Second, the patients were not divided into different clinical OCD subtypes because of the relatively small sample size. There are several clinical subtypes of OCD that depend on the symptom dimensions, such as checking, cleaning, symmetry, and hoarding. Different subtypes may be associated with differential patterns of neural activation [33]. Finally, although no patients had comorbid major depressive disorder, many of the patients with OCD in our study had depressive symptoms. As subjects with concurrent OCD and major depression disorder have different cerebral metabolism compared with those that only have OCD [58], the synchronized baseline brain activity findings in the present study may have been affected by these depressive symptoms.

### Conclusion

In the current study, we analyzed both intra- and inter-regional synchronized and spontaneous brain activity using RS-fMRI in patients with OCD. We found increased local synchronization (i.e., ReHo) of spontaneous brain activity in the OFC, and a negative correlation between local synchronization in the OFC and OCD duration. We also found increased inter-regional synchronized activity (i.e., functional connectivity) between the OFC and vACC. Stronger synchronization of intra- and inter-regional activity in the OFC may have an important role on the pathology of OCD. In addition to orbitofronto-striato-thalamic circuitry, brain regions such as the insula and cerebellum may also be involved in the pathophysiology of OCD.
